# Age-Related Alterations in the Testicular Proteome of a Non-Human Primate

**DOI:** 10.3390/cells10061306

**Published:** 2021-05-24

**Authors:** Jan B. Stöckl, Nina Schmid, Florian Flenkenthaler, Charis Drummer, Rüdiger Behr, Artur Mayerhofer, Georg J. Arnold, Thomas Fröhlich

**Affiliations:** 1 Laboratory for Functional Genome Analysis LAFUGA, Gene Center, LMU München, 81377 München, Germany; stoeckl@genzentrum.lmu.de (J.B.S.); flenkenthaler@genzentrum.lmu.de (F.F.); 2 Biomedical Center (BMC), Anatomy III–Cell Biology, Medical Faculty, LMU München, 82152 Martinsried, Germany; nina.schmid@bmc.med.lmu.de (N.S.); mayerhofer@bmc.med.lmu.de (A.M.); 3 Platform Degenerative Diseases, German Primate Center, Leibniz Institute for Primate Research, 37077 Göttingen, Germany; cdrummer@dpz.eu (C.D.); rbehr@dpz.eu (R.B.); 4 DZHK (German Center for Cardiovascular Research), Partner Site Göttingen, 37077 Göttingen, Germany

**Keywords:** ageing, testis, proteome, marmoset monkey, non-human primate, testicular aging

## Abstract

Aging of human testis and associated cellular changes is difficult to assess. Therefore, we used a translational, non-human primate model to get insights into underlying cellular and biochemical processes. Using proteomics and immunohistochemistry, we analyzed testicular tissue of young (age 2 to 3) and old (age 10 to 12) common marmosets (*Callithrix jacchus*). Using a mass spectrometry-based proteomics approach, we identified 63,124 peptides, which could be assigned to 5924 proteins. Among them, we found proteins specific for germ cells and somatic cells, such as Leydig and Sertoli cells. Quantitative analysis showed 31 differentially abundant proteins, of which 29 proteins were more abundant in older animals. An increased abundance of anti-proliferative proteins, among them CDKN2A, indicate reduced cell proliferation in old testes. Additionally, an increased abundance of several small leucine rich repeat proteoglycans and other extracellular matrix proteins was observed, which may be related to impaired cell migration and fibrotic events. Furthermore, an increased abundance of proteins with inhibitory roles in smooth muscle cell contraction like CNN1 indicates functional alterations in testicular peritubular cells and may mirror a reduced capacity of these cells to contract in old testes.

## 1. Introduction

In many organs, aging is associated with structural alterations and reduced functionality [1,2]. There is increasing evidence that aging of the human testis results in impairment of the two testicular main functions: steroidogenesis and spermatogenesis. More specifically, it has been observed that testicular aging leads to reduced levels of testosterone, a hormone essential for spermatogenesis [3], to an increase of de novo mutations in germ cells [4], alterations in sperm DNA methylation [5] and to lower gamete quality [6,7]. Additionally, there is evidence for structural alterations such as enlargement and sclerosis of the peritubular wall [8,9,10]. However, this was disputed by a recent study, which did not find any alterations in the peritubular wall [11]. This study rather reported a reduced spermatogenic efficiency, increased number of proliferating A_dark_ spermatogonia and increased size of Sertoli cell nucleus and nucleolus. Another recent study reported a decline of both Sertoli and Leydig cells in older men and an unchanged steroidogenic capacity of Leydig cells [10].

Although the described age-related alterations do not always negatively influence fertility, gradual changes of testicular morphology and a decline in fertility starting in the thirties are well documented [12,13,14,15]. However, despite evidence of functional and morphological changes in the aged testis, the topic remains controversial and underlying molecular mechanisms are not yet completely understood. This is in part due to the difficulty to distinguish the sole contribution of age (healthy aging) to the reported morphological and molecular alterations from other parameters, namely medical history and lifestyle. 

These limitations are considerably reduced by studying testicular aging in an adequate model. The non-human primate *C. jacchus* qualifies as a model organism because these animals can be kept under a well-defined environment, and testicular tissue of young healthy individuals is available. Two recent studies [16,17] examined testicular peritubular cells (TPCs) from *C. jacchus* (MKTPCs). TPCs transport sperm, secrete factors and are closely associated with the basal lamina of the germinal epithelium, thereby contributing to the spermatogonial stem cell niche and to the testicular paracrine signaling network [18,19,20,21,22,23,24,25]. It became evident that MKTPCs are very closely related to human TPCs (HTPCs), indicated by the vast similarities in proteomes and expression of markers [16].

TPCs from men and *C. jacchus* were also employed to study aspects of cellular aging [17,23]. In both studies, cells were repeatedly passaged until a state of replicative senescence was reached. Replicative senescence is considered a hallmark of aging [1,26] and is characterized by a stable growth cycle arrest, increased cell size, expression of senescence-associated beta galactosidase secretion and the development of senescence-associated secretory phenotype (SASP) [2,27]. The proteomes of repeatedly passaged MKTPCs were analyzed and compared with the results of MKTPCs isolated from young animals and older animals [17]. Repeated passaging led to alterations indicating cellular senescence of TPCs, impaired protein secretion and a decrease of proteins associated with peritubular wall contractility. The alterations between MKTPCs isolated directly from young and older individuals were, in general, of similar nature but changes in abundance were far more subtle [17].

Besides TPCs, other testicular cell types may age and contribute to age-related alterations of testicular function. To investigate this in a comprehensive manner and to explore healthy aging, we performed a holistic proteome analysis to compare testicular tissues from young and old individuals. We chose a mass spectrometry-based proteomics approach, because of its capability to identify and quantify thousands of individual proteins, facilitating a comprehensive overview of proteome alterations between the testis of young and older individuals. The study was complemented by immunohistochemical studies.

## 2. Materials and Methods

### 2.1. Animals

Testicular tissue from common marmoset monkeys (*Callithrix jacchus*) was examined in this study. The tissue was collected from animals from a self-sustaining colony at the German Primate Center (Deutsches Primatenzentrum–Leibniz-Institute for Primate Research, DPZ, Göttingen, Germany) as described previously [16,17]. 

The legal and the institutional guidelines of the DPZ for the care and use of marmoset monkeys were followed. Health and well-being of the animals were controlled daily by experienced animal care attendants and regularly, at least twice per week, by veterinarians. Marmoset monkeys are social tree-living New World monkeys originating from the tropical north-east of Brazil. Accordingly, the animals were pair-housed in a temperature- (25 ± 1 °C) and humidity-controlled (65 ± 5%) facility. These parameters were controlled daily. Room air was changed several times per hour and filtered adequately. Illumination was provided by daylight and additional artificial lighting on a 12 h/12 h light/dark cycle. Each cage consisting of stainless steel had a vertical orientation (165 cm (height) × 65 cm (width) × 80 cm (depth)) and was furnished with wooden branches and shelves for environmental enrichment and contained a wooden sleeping box simulating the monkeys’ natural habitats. The housing room and the cages were cleaned with water at weekly intervals. The animals were fed ad libitum with a pelleted marmoset diet (ssniff Spezialdiäten, Soest, Germany). In addition, 20 g mash per animal was served in the morning and 30 g fruits or vegetables mixed with noodles or rice were supplied in the afternoon. Furthermore, once per week mealworms or locusts were served. Drinking water was always available. All materials were changed regularly, cleaned, and sterilized.

The “young” group within this study included 6 adult, healthy, sexually mature [28] animals aged on average 2.8 ± 0.6 (SD) years (median = 2.7 years) (2 years (yrs) 2 month (mo), 2 yrs 3 mo, 2 yrs 8 mo, 2 yrs 9 mo, 3 yrs, 3 yrs 10 mo). The “old” group comprised 4 healthy animals aged on average 11.2 ± 0.7 (SD) years (median = 11.5 years) (10 yrs 2 mo, 11 yrs 4 mo, 11 yrs 7 mo, 11 yrs 8 mo). At the age of 8 years, animals can be regarded as old [29]. Three of the animals in the “young” group were euthanized in February, one in April and two in June. Three of the animals in the “old” group were euthanized also in February and one animal in October. The old animals were proven fathers and kept with a female. The young animals were never used for breeding purposes, but showed, like the old ones, qualitatively full (histologically normal) spermatogenesis. The young animals were mostly kept in male-male pairs. All organs were obtained after euthanasia. Euthanasia and testicular tissue removal were carried out by experienced veterinaries according to the institutional guidelines of the DPZ for the care and use of marmoset monkeys according to the applicable law (German Animal Protection Act). One part of each testis was snap frozen in liquid nitrogen immediately upon isolation and used for mass spectrometry analysis and the other part was fixed in Bouin’s Solution and embedded in paraffin for further sectioning and immunohistochemistry.

### 2.2. Immunohistochemistry

Immunohistochemistry was carried out as described earlier [30]. In brief, sections (6 µm) were dewaxed and incubated with the following antibodies: calponin-1 (C-term) (CNN1) monoclonal (1:250; #1806-1, Epitomics, Cambridge, UK), collagen type I (COL1A1) polyclonal (1:1000; OriGene, Rockville, MD, USA), osteoglycin (G-1) (OGN) monoclonal (1:200; sc-374463, Santa Cruz Biotechnology, Inc., Dallas, TX, USA). The nuclei were counterstained with haematoxylin (Carl Roth, Karlsruhe, Germany). Images were taken with a Zeiss Axioplan microscope (Carl Zeiss, Oberkochen, Germany) and digitalized (Jenoptik, Jena, Germany). Per age group sections of at least four monkeys were stained. Controls consisted of omission of the primary antibody.

### 2.3. Measurement of Tubular Diameter

Small testicular sections from the same testes, which were also analyzed by mass spectrometry, were stained with haematoxylin and eosin (HE) (Carl Roth, Karlsruhe, Germany), images were taken with a Zeiss Axioplan microscope (Carl Zeiss, Oberkochen, Germany) and digitalized (Jenopitk, Jena, Germany). Only those tubules, which were cross sectioned and showed a roundish appearance, were analyzed. The smallest and the largest diameters were determined using Fiji software. The means of the largest and smallest, as well as only the smallest diameter, were then used for statistical analysis. Per animal, the diameter of at least ten randomly selected tubuli were determined, in total 50 per group (young group: *n* = 5; old group: *n* = 4). For statistical analysis, an unpaired t-test was performed using Prism 6 (GraphPad, San Diego, CA, USA). In addition, we analyzed sections from HE stained samples used in a previous publication [16]. They stem from two young (2 and 3 years) and two old (9 and 12 years) *C. jacchus*. Tubular diameters (young: *n* = 138, old: *n* = 153) were evaluated as described above. For statistical analysis, a one-way ANOVA following a Tukey´s multiple comparisons test was used.

### 2.4. Sample Preparation for LC-MS/MS

Testicular proteomes of young (*n* = 6) and old (*n* = 4) animals were analyzed. Then, 100 µL of lysis buffer (8 M of urea in 50 mM ammonium bicarbonate) was added to approximately 1 mg of testicular tissue. For lysis and homogenization, samples were sonicated using a cup resonator (Bandelin, Berlin, Germany) and further processed with QIAshredder (QIAGEN, Hilden, Germany) centrifugation devices (4 °C, 2500× *g*, 1 min). Protein concentration was determined with a Pierce 660 nm assay (Thermo Scientific, Waltham, MA, USA). Then, 20 µg of protein were reduced in 4 mM dithiothreitol/2 mM tris(2-carboxyethyl)phosphine and subsequently alkylated in 8 mM iodoacetamide. Proteins were digested in two steps: (i) Lys-C (1/100, enzyme/protein, FUJIFILM Wako, Neuss, Germany) for 4 h and (ii) trypsin (1/50 enzyme/protein, Promega) overnight at 37 °C.

### 2.5. LC-MS/MS Analysis

LC-MS/MS analysis was carried out on an Ultimate 3000 RSLC coupled with a Q Exactive HF-X (both Thermo Scientific, Waltham, MA, USA). Aliquots of 1.5 µg peptides were injected onto a trap column (PepMap 100 C18, 100 μm × 2 cm, 5 μM particles, Thermo Scientific, Waltham, MA, USA) at a flow rate of 5 μL/min mobile phase A (0.1% formic acid and 1% acetonitrile in water). Peptide separation was performed with an EASY-Spray column (PepMap RSLC C18, 75 μm × 50 cm, 2 μm particles, Thermo Scientific, Waltham, MA, USA) and a flow rate of 250 nL/min. A two-step gradient was used: first from 3% mobile phase B (0.1% formic acid in acetonitrile) to 25% B in 160 min and second a 10 min ramp to 40% B (A: 0.1% formic acid in water). Peptides were analyzed in the data-dependent acquisition mode with up to 15 MS/MS scans per cycle. 

### 2.6. Data Analysis

Acquired MS spectra were searched using MaxQuant (1.6.5.0) with the match between runs feature activated and quantified using the label-free quantification approach [31]. Amino acid sequences were retrieved from UniProt using all available entries for *C. jacchus* from both Swiss-Prot and TrEMBL (retrieval: 09/2020). Data analysis was done with Perseus (1.6.5.0) and R (4.0.1) [32]. Volcano plot analysis, principal component analysis (PCA) as well the heatmap were performed with the built-in features of Perseus. For multiple testing correction, a significance cut-off curve was generated (s0 = 0.1, FDR < 0.05) [33]. Differentially abundant proteins were annotated with the PANTHER online tool using Gene Ontology (GO) biological process as a database [34]. Proteins significantly altered in abundance were further analyzed and annotated using DAVID and STRING [35,36,37]. For the DAVID analysis, the functional annotation clustering tool was used with the following categories: GO biological process, GO cellular component, GO molecular function, Reactome, UniProt keyword entries. Classification stringency was set to high and resulting clusters were labeled according to the term with the smallest *p*-value. For the STRING analysis, all interactions but neighborhood and gene fusion were included, and nodes without significant interaction were removed. The mass spectrometry proteomics data were deposited to the ProteomeXchange Consortium (http://proteomecentral.proteomexchange.org, accessed on 23 May 2021) via the Proteomics Identification Database (PRIDE) partner repository with the dataset identifier PXD024844 [38].

## 3. Results

### 3.1. Histology Reveals Ongoing Spermatogenesis in All Samples and Slightly Increased Tubular Diameters in Old Animals

**Figure 1:**
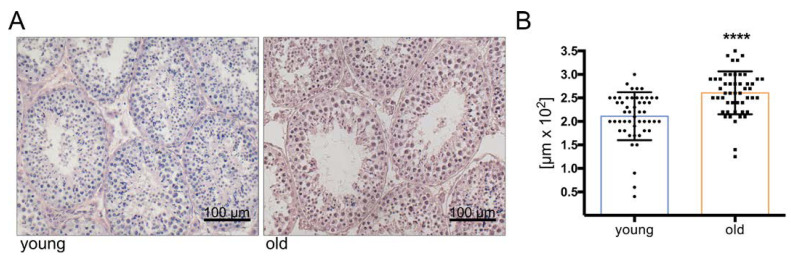
Light micrograph of HE stained sections of testes from young (3 years, left) and old *C. jacchus* (11 years, right). (**A**) Scale bar indicates 100 µm. Tubular diameters of young (2–3 years, *n* = 5) and old (10–12 years, *n* = 4) *C. jacchus.* (**B**) Tubular diameter was slightly increased in the old *C. jacchus* (**** *p <* 0.0001; unpaired *t*-test); individual diameters are shown, and bars indicate the mean and SD.

**Figure A1:**
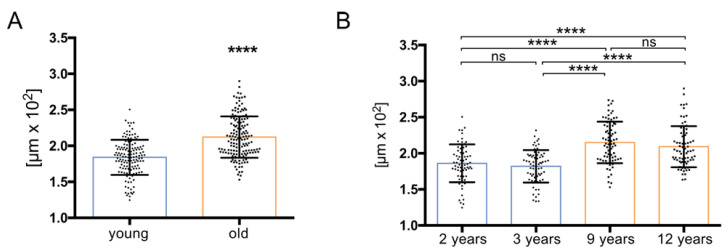
Tubular diameter of young (2 and 3 years, total *n* = 138 tubules) and old (9 and 12 years, total *n* = 153 tubules) *C. jacchus* (**A**). Tubular diameter was slightly increased in old *C. jacchus* (**** *p* < 0.0001; unpaired *t*-test). Tubular diameter of individual *C. jacchus* (**B**). (young (2 years), *n* = 69 tubules; young (3 years), *n* = 69 tubules; old (9 years), *n* = 77 tubules and old (12 years), *n* = 76 tubules). Statistical analysis: one-way ANOVA following Tukey´s test (ns *p* > 0.05; **** *p* < 0.0001). Individual diameters are shown, and bars indicate the mean and SD.

Histological evaluation of testis sections from six young (2 to 3 years) and four old (10 to 12 years) monkeys indicated ongoing spermatogenesis in all specimens (Figure 1). Due to the handling of the testes, germ cells were occasionally detached and were observed in the lumen. However, measurements of tubular diameters were possible and revealed slightly increased diameters in the older individuals (*p* < 0.05) (Figure 1A). The increase was significant, irrespective of whether only the smallest diameter (Figure 1B) or the mean of smallest and largest diameters (not shown) were taken into consideration. Similar increases were found in additional sections from young and old monkeys (Figure A1).

### 3.2. Comprehensive Proteome Analysis of Testes of Young and Old Individual Donors

The proteomes of testicular tissue derived from six young (age: on average 2.8 years old) and four old (age: on average 11.2 years old) *C. jacchus* individuals were analyzed by LC MS/MS leading to the identification of 63,510 peptides and 5924 proteins (Table S1). Several of the identified proteins are characteristic of specific testicular cell types (Table 1). For instance, ATP-dependent RNA helicase DDX4 (DDX4) and melanoma-associated antigen 4 (MAGEA4) are in the testis specific for germ cells [39,40], cholesterol side-chain cleavage enzyme, mitochondrial (CYP11A1) and insulin-like 3 (INSL3) are markers specific for Leydig cells [41,42]. Furthermore, cyclin-dependent kinase inhibitor 1B (CDKN1B) and aortic smooth muscle actin (ACTA2, also referred to as alpha-smooth muscle actin) are common markers for adult Sertoli cells and peritubular cells, respectively [19,43]. Additionally, vimentin (VIM) is common in somatic testicular cell types such as peritubular cells, Sertoli cells and interstitial cells [44].

**Figure 2:**
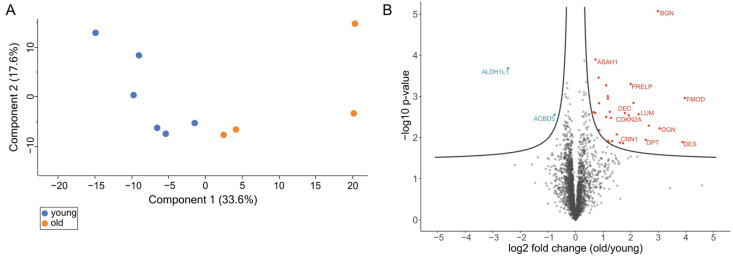
(**A**) Principal component analysis of old (red dots) and young (blue dots) *C. jacchus* testicular proteome profiles. Numbers in parentheses indicate the percentage of variation each component explains. (**B**) Volcano plot of old vs. young testis proteomes. Significance cut off curve was generated using the parameters FDR < 0.05 and s_0_ = 0.1. Proteins above the curve are considered significant; red color indicates higher abundance in old donors; blue color indicates lower abundance in old donors.

**Figure A2:**
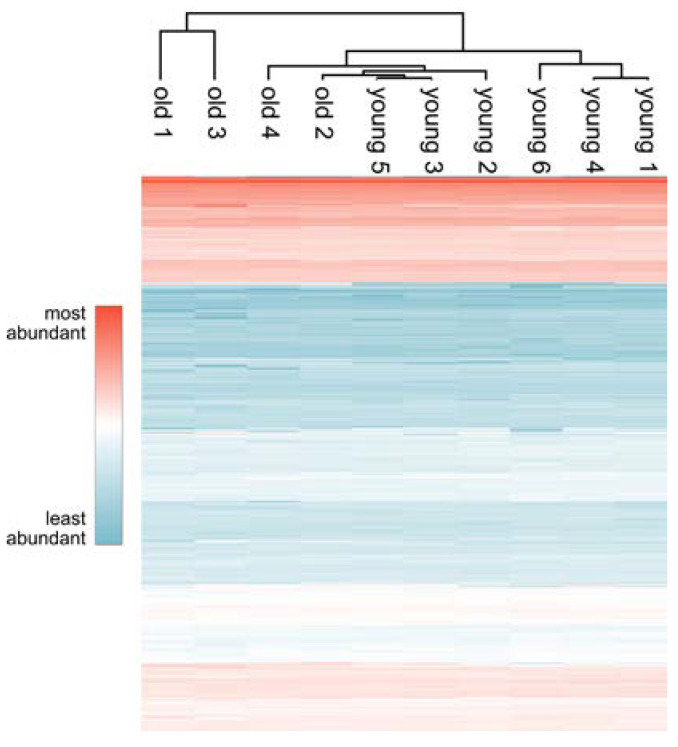
Unsupervised hierarchical clustering of old and young *C. jacchus* testicular proteome profiles.

Proteins were quantified using a label-free quantification approach and filtered with the criteria: having at least 90% valid values among either old or young samples. This resulted in 3217 sufficiently quantified proteins. The quantitative data were analyzed using unsupervised hierarchical clustering and principal component analysis (PCA) (Figure 2A and Figure A2). PCA shows a separation of testes proteome profiles from old and young donors (Figure 2A). In contrast, the heatmap shows three main clusters (Figure A1). While proteome profiles from two old individuals are separated from those of the other eight, one cluster contains three young and two old individuals and a third cluster three samples from young individuals.

### 3.3. Volcano Plot Analysis Reveals Significant Alterations in the Testis Proteome of Older Individuals

**Figure 3:**
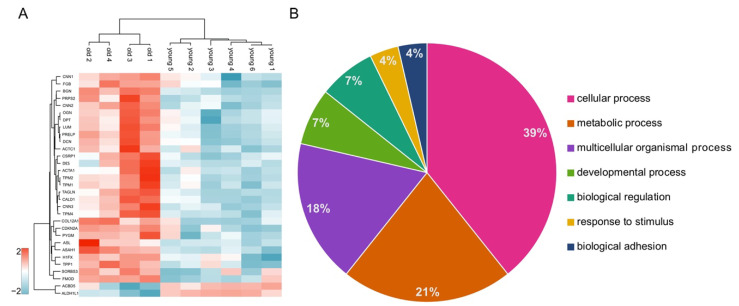
(**A**) Heatmap and unsupervised hierarchical clustering of differentially abundant proteins for the comparison old vs. young testicular proteomes. Data were z-scored prior to the analysis. (**B**) PANTHER analysis of differentially abundant proteins using GO biological process as database. The numbers indicate the percentage of proteins annotated with the corresponding GO term.

A volcano plot analysis revealed 31 differentially abundant proteins in the testicular proteomes of the older individuals compared to the proteomes of the young testes (Figure 2B, Table S2) (FDR < 0.05). All these proteins were clustered in a z-score normalized heatmap, illustrating the strong differences in abundance (Figure 3A). Furthermore, a higher variance in the abundance of these proteins within the samples from old individuals compared to samples from the young individuals became apparent. Conspicuously, only two of the differentially abundant proteins were less abundant in the testis samples of aged animals: (i) cytosolic 10-formyltetrahydrofolate dehydrogenase (ALDH1L1) and (ii) acyl-CoA-binding domain-containing protein 5 (ACBD5). The 29 more abundant proteins could predominantly be assigned to three protein families: four proteins from the actin-binding calponin repeat family, three tropomyosins and several members of the small leucine-rich proteoglycans (SLRPs) A Gene Ontology analysis using the PANTHER online tool was used to classify the differentially abundant proteins (Figure 3B).

### 3.4. DAVID Analysis Reveals Clusters of Enriched Functional Terms

**Figure 4:**
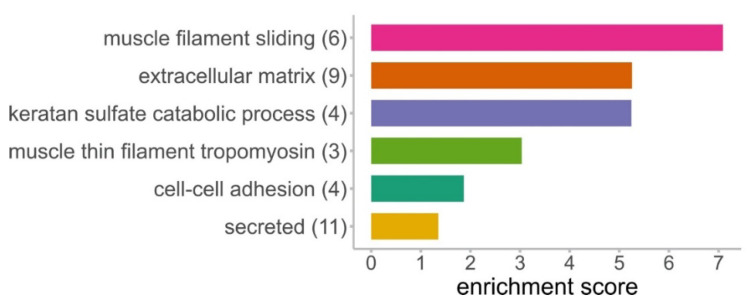
Clusters of functionally enriched terms generated from proteins differentially abundant in testes of old donors. Numbers in brackets indicate the number of identified proteins in one cluster. Categories used for the DAVID tool were: GO biological process, GO cellular component, GO molecular function, Reactome, UniProt keywords.

The data were annotated and analyzed with DAVID resulting in functionally enriched terms which were subsequently clustered (Figure 4, Table S2). Six clusters were found to be significantly enriched, and the most significant term was used to label the cluster. Out of the 31 differentially abundant proteins, 21 are represented in at least one of these clusters. The cluster named “muscle filament sliding” showed the highest enrichment score and comprises desmin (DES), actins and three tropomyosins. These three tropomyosins were also found in the cluster “muscle thin filament tropomyosin”. In the second most enriched cluster “extracellular matrix”, all above mentioned SLRPs, collagen alpha-1(XII) chain (COL12A1), dermatopontin (DPT) and fibrinogen beta chain (FGB) were found. The cluster “keratan sulfate catabolic process” contained four differentially abundant SLRPs: lumican (LUM), fibromodulin (FMOD), mimecan (OGN) and prolargin (PRELP). However, in our dataset, no enzyme of this pathway was identified. The cluster “cell-cell adhesion” comprises actin-binding proteins like calponins and caldesmon (CALD1). The cluster “secreted” includes enzymes like tripeptidyl-peptidase 1 (TPP1) and acid ceramidase (ASAH1) in addition to the proteins of the cluster “extracellular matrix”.

### 3.5. STRING Analysis Results in a Rich Interaction Network

**Figure 5:**
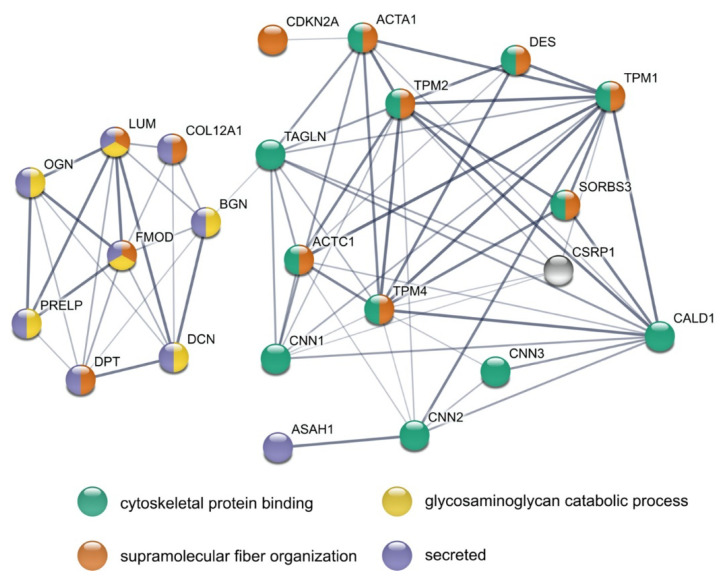
STRING analysis of all differentially abundant proteins in the testis of old vs. young donors. Nodes represent individual proteins and are colored based on the four annotations indicated at the bottom of the figure. Line strength indicates confidence level from low (0.150) to highest (0.900).

The 31 differentially abundant proteins were analyzed with STRING (Figure 5). This resulted in significantly more interactions than expected by chance (PPI enrichment *p*-value: 1.0 × 10^−16^, expected number of edges: 5, number of edges found: 73). From 31 proteins, 23 had at least one interaction with another differentially abundant protein. Strikingly, two distinct networks can be observed, of which the smaller contained all the differentially abundant SLRPs together with DPT and COL12A1. All of them are annotated to be secreted, and some are known to be involved in “supramolecular fiber organization”, while others are part of “glycosaminoglycan catabolic process”. LUM and FMOD are part of both gene sets. The other network comprises various cytoskeletal proteins, of which around half are also known to be involved in “supramolecular fiber organization”. Cysteine and glycine-rich protein 1 (CSRP1) is not annotated in the four chosen categories but is, e.g., involved in actin cytoskeleton organization.

### 3.6. Immunostaining Illustrates the Increased Abundance of Extracellular Matrix Proteins

**Figure 6:**
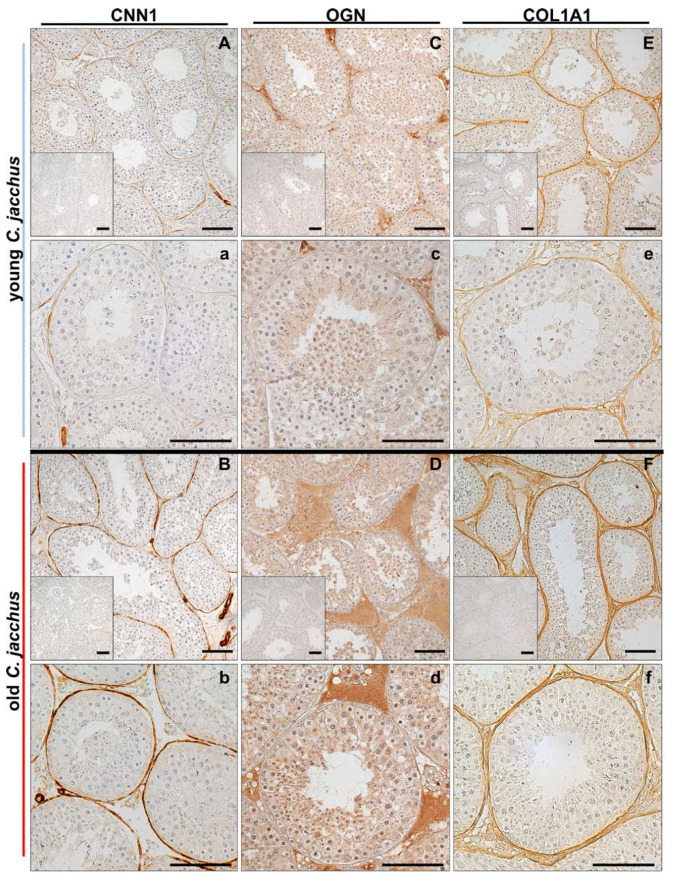
Representative light micrographs of immunohistochemistry. Testicular sections from young (2–3 years) and old (10–12 years) *C. jacchus* were stained with specific antibodies against CNN1, OGN and COL1A1. CNN1 staining was found in most peritubular cells from young *C. jacchus* (**A**,**a**, (higher magnification)). In old *C. jacchus,* CNN1 was expressed in all peritubular cells (**B**,**b** (higher magnification)). OGN was detected mainly within the interstitial space of testes of young *C. jacchus* (**C**,**c** (higher magnification)). The level increased in the testes of old *C. jacchus* (**D**,**d** (higher magnification)). COL1A1 was detected in peritubular cells from young (**E**,**e** (higher magnification)) and old (**F**,**f** (higher magnification)) *C. jacchus.* In old individuals, COL1A1 expression increased in the interstitial space. Nuclei were slightly stained with hematoxylin. Inserts: negative controls. Scale bars = 100 µm.

Immunohistochemistry revealed expression of CNN1 in testicular peritubular cells of young and old *C. jacchus* (Figure 6A,a,B,b). Smooth muscle cells of testicular blood vessels were also immunopositive for CNN1 and served as internal staining control. In the young individuals, not all peritubular cells were immunopositive. In contrast, in the old individuals all peritubular cells were stained. 

OGN was detected mainly within the interstitial space of both, young and old individuals (Figure 6C,c,D,d). More intensive staining of larger interstitial areas indicates higher abundance of OGN. Both findings nicely corroborate our proteome analysis, where CNN1 (log2 fold change: 1.61; *p*-value: 0.013) and OGN (log_2_ fold change: 3.05; *p*-value: 0.0060) were found to be more abundant in the old testes.

Collagen alpha-1(I) chain (COL1A1) was localized in the peritubular wall compartment and the interstitial space, with higher abundance in old individuals (Figure 6E,e,F,f,). In the proteome analysis, a trend (*p*-value: 0.14; log_2_ fold change: 0.72) towards an increased COL1A1 abundance was found.

## 4. Discussion

### 4.1. General Remarks

This study was performed to examine age-related changes in testicular proteins. We analyzed the testicular proteomes of six young (2 to 3 years) and four old (10 to 12 years) *C. jacchus* individuals. Captive marmosets show the first signs of aging around the age of 8 years, and therefore, the older animals can be regarded as old, healthily aged individuals [29,45,46]. We were able to directly compare testicular tissues from young and old individuals grown up under well-characterized conditions in the same colony and thereby minimizing influences of lifestyle. Using a bottom-up mass spectrometry approach, we identified 5924 proteins, among them several specific for germ cells and somatic cells, like Leydig and Sertoli cells, in the testis. This indicates a profound analytical depth, shows that all major testicular cell types are captured by our analysis and thus demonstrates the relevance of our dataset. Principal component analysis (PCA) of LFQ intensity values led to a clear separation between old and young testicular proteomes, indicating significant alterations between the testis proteomes of young and old donors (Figure 2A). Further statistical evaluation revealed 31 differentially abundant proteins. The large majority (29 proteins) was more abundant in the testes of old animals (Figure 2B). Strikingly, most of these proteins belong to just a few enriched protein families and categories (Figure 3B, Figure 4 and Figure 5). The abundance of common Sertoli and Leydig cell markers [10], as well as of germ cell-specific proteins, was not altered in older animals. This result is in line with the observed ongoing spermatogenesis in both age-groups.

### 4.2. Levels of Anti-Proliferative Proteins Are Increased in Testes from Older Individuals

Among proteins more abundant in old testes are various proteins known to inhibit cell proliferation. The most prominent one is CDKN2A (log2 fold change: 1.92; *p*-value: 0.0029), also called p16^INK4a^, which is a strong interactor of cyclin dependent kinase 4 and 6 [47]. This interaction directly regulates the transition of a cell from G1 phase into S1 phase [47]. Therefore, an increase of this protein is typically associated with a growth cycle arrest, a characteristic attribute of cellular senescence [48,49]. Additionally, in skin, CDKN2A has been described as an in vivo biomarker for cellular senescence since its expression in dermis and epidermis cells positively correlates with chronological aging [50]. We attempted to localize this protein in sections of testes by immunohistochemistry, yet the findings did not yield specific results. Hence the nature of cells, in which CDKN2A increases, remains to be defined.

Furthermore, three extracellular proteins increased in old testes, show anti-proliferative activity, namely, the two SLRPs PRELP and OGN as well as DPT. PRELP is known to be typically located near basement membranes and is supposed to act as an anchor to connective tissue [51]. The inhibitory effect of PRELP on proliferation was recently shown for hepatocellular carcinoma cells but otherwise its biological function is not well known [52]. Another extracellular protein known to play a role in inhibition of proliferation is DPT, which is involved in the organization of collagen fibrils in the extracellular matrix [53,54]. Additionally, it was demonstrated that an increased abundance of DPT is associated with testicular dysfunction [54,55,56]. OGN was shown to inhibit proliferation in cardiac smooth muscle tissue and influences apoptosis and cell migration [57,58]. A strong increase of OGN in old testes was also shown by immunostaining. OGN was predominantly found in the interstitial compartment, which contains extracellular matrix and fibroblasts, next to Leydig cells, macrophages and blood vessels. Taken together, the alterations in proteins regulating cell proliferation indicate that mitotic events in old testes are slowed down or even arrested.

Associated with testicular dysfunction is the enzyme ASAH1, which was increased in old testes. ASAH1 is a regulator of steroidogenic capacity by controlling the turnover of ceramide and sphingosine. It may also influence steroidogenesis directly by binding to NR5A1 [59,60]. Its substrate, ceramide, is a bioactive lipid which regulates apoptosis, differentiation but also proliferation [61,62]. In human ovarian granulosa cells, it induced cell death [63].

### 4.3. Testis Proteomes of Aged Individuals Show a Broad Increase of Actin-Binding Proteins

The proteomics analysis showed an increase of actin-binding proteins in the old testes, including all three calponin isoforms. One of them, calponin-1 (CNN1) is specific for smooth muscle cells, a fact reflected by the immunostaining of testicular peritubular cells and typical smooth muscle cells of blood vessels (Figure 6A,a,B,b). Previously, a decline of peritubular cell CNN1 was shown in infertile patients, which went hand in hand with a partial loss of other contractility proteins of peritubular cells and indicated loss of the smooth muscle phenotype, in general [64]. In the present study, stronger staining of CNN1 in peritubular cells of old testes was observed, whereas in young testes not all peritubular cells were positive for CNN1. This implies that CNN1 expression increases with advanced age. For ACTA2, another contractility protein [65,66], expression was reported upon onset of puberty in nonhuman primates, but other age-depending changes are not well known. By binding actins, CNN1 plays an important role in the cytoskeleton [67]. Specifically, it has an inhibitory role in smooth muscle contraction and responsiveness regulation [68]. An increase of CNN1 thus may suggest reduced contractile abilities. Calponin-2 (CNN2) also binds actin but has a different functional profile and is involved in cell proliferation, cell motility and immune modulation (reviewed in [68]). Calponin-3 (CNN3) is the least explored. It regulates the actin cytoskeleton and is involved in cell fusion and myogenesis [68]. The functions of CNN2/3 in the testis are not known.

Another more abundant protein in aged testes and a member of the calponin repeat family is transgelin (TAGLN). TAGLN is an actin-binding cytoskeletal protein involved in differentiation, apoptosis and proliferation [69,70,71]. The three isoforms of the actin-binding tropomyosin family, tropomyosin alpha-1 chain (TPM1), tropomyosin beta chain (TPM2) and tropomyosin alpha-4 chain (TPM4), were also more abundant in old testes. Tropomyosins are known to be involved in regulating cell migration and apoptosis (reviewed in [72,73]). Together with the also increased CALD1, they regulate smooth muscle contraction and contribute to the stabilization of the cytoskeleton [74]. By interacting with tropomyosins, CALD1 inhibits the contraction of smooth muscle cells, and an increase of CALD1 is associated with a higher inhibitory effect [75,76,77].

Several actin-binding cytoskeletal proteins, including CNN2, TAGLN, CALD1 and TPM1, which were more abundant in old testes, also have anti-proliferative functions. CNN2 and CALD1 are linked to inhibition of proliferation in smooth muscle cells [78,79,80]. For CNN2, this effect was also shown in fibroblasts and prostate cancer cells [81]. Furthermore, the potential involvement of TPM1 in the inhibition of cancer and vascular smooth muscle cell proliferation has been demonstrated [82,83,84].

Taken together, the increase of several actin-binding proteins, specifically CNN1, CALD1, tropomyosins, which can inhibit smooth muscle cell proliferation and contraction, may indicate a reduced capacity of cells, presumably peritubular cells and typical smooth muscle cells of blood vessels, to contract in aged testes.

### 4.4. Proteomic Alterations in Old Testes Point to Specific Age-Related Changes of Peritubular Cells

TPCs are smooth muscle-like cells, which form a small compartment, the peritubular wall of the seminiferous tubules. They are able to contract and thereby crucial for sperm transport [25]. They also secrete extracellular matrix components and various signaling factors [19,20]. Importantly, they express several of the above discussed proteins, namely, CNN1, CNN2, TPM1, TAGLN and CALD1. 

Replicative senescence of isolated TPCs from *C. jacchus* has recently been studied at the proteome level and furthermore we compared TPCs isolated from old and young animals. The results revealed alterations of proteins related to impairments of protein secretion and, of note, reduced levels of proteins related to contractility [17]. The proteins identified include CNN1, MYH11, ACTA2 and DES. Hence, both studies, addressing either whole testes or isolated TPCs, evidence a reduction in contractility-associated proteins. This points to reduced contractile abilities of TPCs in old animals.

In this context, the slight but significantly increased diameter of the seminiferous tubules in old animals is of note (Figure 1). It may indicate a reduced contractile state or tone of peritubular cells but could be due to other reasons as well, including alterations in the composition of the tubular wall. The immunostaining showed an increase of COL1A1 in the peritubular wall in older testes. Increased abundance of collagen is associated with fibrosis, [85], which is typically observed in the testes of infertile men [86,87,88]. Such changes could potentially impair the ability to contract in the old testes, as well. Fibrosis, including the interstitial areas, is indicated by higher levels of SLRPs, namely, decorin (DCN), biglycan (BGN), fibromodulin (FMOD) and lumican (LUM), which are involved in the maintenance of collagen I fibrils [89,90,91,92] and might play a role in the observed alterations of the peritubular wall. The function of FMOD and LUM in the testis is unknown, but DCN and BGN are known products of TPCs [93,94]. They serve structural roles but can also interfere with signaling factors and receptors, as shown in HTPCs [95]. Increased amounts thus imply that the local signaling in the testis and functions of TPCs, in particular, may be impaired.

Whether and to what degree smooth muscle cells of blood vessels, present in whole testes, may contribute to the proteomic changes remains to be studied. This warrants additional studies to address the question, whether aging of the testes may include alterations of blood vessels and hence blood flow.

## 5. Conclusions

To investigate the mechanisms which underly healthy testicular aging in a translational non-human primate model we performed a proteome analysis complemented by immunohistochemistry. Our data demonstrate that testicular aging is associated with proteome alterations, including increased levels of a variety of anti-proliferative proteins. Furthermore, several proteins which can impair smooth muscle cell contraction and extracellular matrix proteins were more abundant in old testes. They point to age-associated changes specifically in smooth muscle cells and smooth-muscle-like cells of the peritubular wall. While proteomics is a powerful tool to gain new insights into complex biochemical networks, additional studies are now necessary to obtain mechanistic insights.

## Figures and Tables

**Table 1:** Overview of identified markers for different testicular cell types.

Cell Type	Markers
Leydig cells	CYP11A1, CYP17A1, INSL3
Sertoli cells	VIM, CDKN1B
Peritubular cells	VIM, ACTA2
Germ cells	DDX4, MAGEA4, UTF1

## Data Availability

The mass spectrometry proteomics data were deposited to the ProteomeXchange Consortium (http://proteomecentral.proteomexchange.org, accessed on 23 May 2021) via the Proteomics Identification Database (PRIDE) partner repository with the dataset identifier PXD024844.

## Supplementary Materials

The following are available online at https://www.mdpi.com/article/10.3390/cells10061306/s1, Table S1: All identified proteins in the proteomes of old and young *Callithrix jacchus* donors; Table S2: Proteins significantly different in abundance in the proteomes of old individuals compared to young individuals. Log2 fold change is old vs. young
